# Mechanism of Salinity Tolerance in Plants: Physiological, Biochemical, and Molecular Characterization

**DOI:** 10.1155/2014/701596

**Published:** 2014-04-03

**Authors:** Bhaskar Gupta, Bingru Huang

**Affiliations:** ^1^Department of Biological Sciences (Section Biotechnology), Presidency University, 86/1 College Street, Kolkata 700073, India; ^2^Department of Plant Biology and Pathology, Rutgers University, New Brunswick, NJ 08901, USA

## Abstract

Salinity is a major abiotic stress limiting growth and productivity of plants in many areas of the world due to increasing use of poor quality of water for irrigation and soil salinization. Plant adaptation or tolerance to salinity stress involves complex physiological traits, metabolic pathways, and molecular or gene networks. A comprehensive understanding on how plants respond to salinity stress at different levels and an integrated approach of combining molecular tools with physiological and biochemical techniques are imperative for the development of salt-tolerant varieties of plants in salt-affected areas. Recent research has identified various adaptive responses to salinity stress at molecular, cellular, metabolic, and physiological levels, although mechanisms underlying salinity tolerance are far from being completely understood. This paper provides a comprehensive review of major research advances on biochemical, physiological, and molecular mechanisms regulating plant adaptation and tolerance to salinity stress.

## 1. Introduction

A major challenge towards world agriculture involves production of 70% more food crop for an additional 2.3 billion people by 2050 worldwide [1]. Salinity is a major stress limiting the increase in the demand for food crops. More than 20% of cultivated land worldwide (~ about 45 hectares) is affected by salt stress and the amount is increasing day by day. Plants on the basis of adaptive evolution can be classified roughly into two major types: the halophytes (that can withstand salinity) and the glycophytes (that cannot withstand salinity and eventually die). Majority of major crop species belong to this second category. Thus salinity is one of the most brutal environmental stresses that hamper crop productivity worldwide [2, 3].

Salinity stress involves changes in various physiological and metabolic processes, depending on severity and duration of the stress, and ultimately inhibits crop production [4–7]. Initially soil salinity is known to represses plant growth in the form of osmotic stress which is then followed by ion toxicity [4, 5]. During the initial phases of salinity stress, water absorption capacity of root systems decreases and water loss from leaves is accelerated due to osmotic stress of high salt accumulation in soil and plants, and therefore salinity stress is also considered as hyperosmotic stress [6]. Osmotic stress in the initial stage of salinity stress causes various physiological changes, such as interruption of membranes, nutrient imbalance, impairs the ability to detoxify reactive oxygen species (ROS), differences in the antioxidant enzymes and decreased photosynthetic activity, and decrease in stomatal aperture [3, 5]. Salinity stress is also considered as a hyperionic stress. One of the most detrimental effects of salinity stress is the accumulation of Na^+^ and Cl^−^ ions in tissues of plants exposed to soils with high NaCl concentrations. Entry of both Na^+^ and Cl^−^ into the cells causes severe ion imbalance and excess uptake might cause significant physiological disorder(s). High Na^+^ concentration inhibits uptake of K^+^ ions which is an essential element for growth and development that results into lower productivity and may even lead to death [4]. In response to salinity stress, the production of ROS, such as singlet oxygen, superoxide, hydroxyl radical, and hydrogen peroxide, is enhanced [8–12]. Salinity-induced ROS formation can lead to oxidative damages in various cellular components such as proteins, lipids, and DNA, interrupting vital cellular functions of plants.

Genetic variations in salt tolerance exist, and the degree of salt tolerance varies with plant species and varieties within a species. Among major crops, barley (*Hordeum vulgare*) shows a greater degree of salt tolerance than rice (*Oryza sativa*) and wheat (*Triticum aestivum*). The degree of variation is even more pronounced in the case of dicotyledons ranging from* Arabidopsis thaliana*, which is very sensitive towards salinity, to halophytes such as* Mesembryanthemum crystallinum*,* Atriplex sp.*,* Thellungiella salsuginea* (previously known as* T. halophila*) [3, 13, 14]. In the last two decades sumptuous amount of research has been done in order to understand the mechanism of salt tolerance in model plant* Arabidopsis* [15]. Genetic variations and differential responses to salinity stress in plants differing in stress tolerance enable plant biologists to identify physiological mechanisms, sets of genes, and gene products that are involved in increasing stress tolerance and to incorporate them in suitable species to yield salt tolerant varieties.

The main aim of this review is to discuss research advances on the complex physiological and molecular mechanisms that are involved in plant salinity tolerance.

## 2. Physiological and Biochemical Mechanisms of Salt Tolerance

Plants develop various physiological and biochemical mechanisms in order to survive in soils with high salt concentration. Principle mechanisms include, but are not limited to, (1) ion homeostasis and compartmentalization, (2) ion transport and uptake, (3) biosynthesis of osmoprotectants and compatible solutes, (4) activation of antioxidant enzyme and synthesis of antioxidant compounds, (5) synthesis of polyamines, (6) generation of nitric oxide (NO), and (7) hormone modulation. Research advances elucidating these mechanisms are discussed below.

### 2.1. Ion Homeostasis and Salt Tolerance

Maintaining ion homeostasis by ion uptake and compartmentalization is not only crucial for normal plant growth but is also an essential process for growth during salt stress [16–18]. Irrespective of their nature, both glycophytes and halophytes cannot tolerate high salt concentration in their cytoplasm. Hence, the excess salt is either transported to the vacuole or sequestered in older tissues which eventually are sacrificed, thereby protecting the plant from salinity stress [19, 20].

Major form of salt present in the soil is NaCl, so the main focus of research is the study about the transport mechanism of Na^+^ ion and its compartmentalization. The Na^+^ ion that enters the cytoplasm is then transported to the vacuole via Na^+^/H^+^ antiporter. Two types of H^+^ pumps are present in the vacuolar membrane: vacuolar type H^+^-ATPase (V-ATPase) and the vacuolar pyrophosphatase (V-PPase) [21–23]. Of these, V-ATPase is the most dominant H^+^ pump present within the plant cell. During nonstress conditions it plays an important role in maintaining solute homeostasis, energizing secondary transport and facilitating vesicle fusion. Under stressed condition the survivability of the plant depends upon the activity of V-ATPase [21]. In a study performed by De Lourdes Oliveira Otoch et al. [22] in hypocotyls of* Vigna unguiculata* seedlings, it was observed that the activity of V-ATPase pump increased when exposed to salinity stress but under similar conditions, activity of V-PPase was inhibited, whereas in the case of halophyte* Suaeda salsa,* V-ATPase activity was upregulated and V-PPase played a minor role [23].

**Figure 1:**
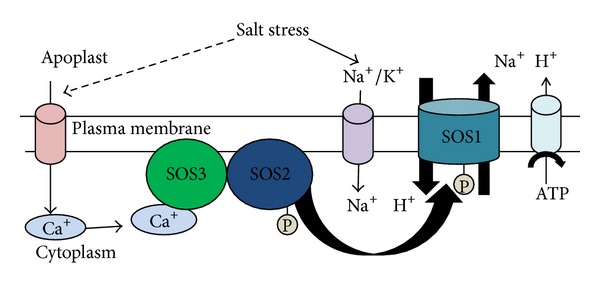
Model of SOS pathway for salinity stress responses.

Increasing evidence demonstrates the roles of a Salt Overly Sensitive (SOS) stress signalling pathway in ion homeostasis and salt tolerance [24, 25]. The SOS signalling pathway (Figure 1) consists of three major proteins, SOS1, SOS2, and SOS3.* SOS1,* which encodes a plasma membrane Na^+^/H^+^ antiporter, is essential in regulating Na^+^ efflux at cellular level. It also facilitates long distance transport of Na^+^ from root to shoot. Overexpression of this protein confers salt tolerance in plants [26, 27].* SOS2* gene, which encodes a serine/threonine kinase, is activated by salt stress elicited Ca^+^ signals. This protein consists of a well-developed N-terminal catalytic domain and a C-terminal regulatory domain [28]. The third type of protein involved in the SOS stress signalling pathway is the SOS3 protein which is a myristoylated Ca^+^ binding protein and contains a myristoylation site at its N-terminus. This site plays an essential role in conferring salt tolerance [29]. C-terminal regulatory domain of SOS2 protein contains a FISL motif (also known as NAF domain), which is about 21 amino acid long sequence, and serves as a site of interaction for Ca^2+^ binding SOS3 protein (Figure 1). This interaction between SOS2 and SOS3 protein results in the activation of the kinase [30]. The activated kinase then phosphorylates SOS1 protein thereby increasing its transport activity which was initially identified in yeast [31]. SOS1 protein is characterised by a long cytosolic C-terminal tail, about 700 amino acids long, comprising a putative nucleotide binding motif and an autoinhibitory domain. This autoinhibitory domain is the target site for SOS2 phosphorylation (Figure 1). Besides conferring salt tolerance it also regulates pH homeostasis, membrane vesicle trafficking, and vacuole functions [32, 33]. Thus with the increase in the concentration of Na^+^ there is a sharp increase in the intracellular Ca^2+^ level which in turn facilitates its binding with SOS3 protein. Ca^2+^ modulates intracellular Na^+^ homeostasis along with SOS proteins. The SOS3 protein then interacts and activates SOS2 protein by releasing its self-inhibition. The SOS3-SOS2 complex is then loaded onto plasma membrane where it phosphorylates SOS1 (Figure 1). The phosphorylated SOS1 results in the increased Na^+^ efflux, reducing Na^+^ toxicity [34].

Many plants have developed an efficient method to keep the ion concentration in the cytoplasm in a low level. Membranes along with their associated components play an integral role in maintaining ion concentration within the cytosol during the period of stress by regulating ion uptake and transport [35]. The transport phenomenon is carried out by different carrier proteins, channel proteins, antiporters and symporters. Maintaining cellular Na^+^/K^+^ homeostasis is pivotal for plant survival in saline environments. Ma et al. [36] have reported that Arabidopsis NADPH oxidases* AtrbohD* and* AtrbohF* function in ROS-dependent regulation of Na^+^/K^+^ homeostasis in Arabidopsis under salt stress. Plants maintain a high level of K^+^ within the cytosol of about 100 mM ideal for cytoplasmic enzyme activities. Within the vacuole K^+^ concentration ranges between 10 mM and 200 mM. The vacuole serves as the largest pool of K^+^ within the plant cell. K^+^ plays a major role in maintaining the turgor within the cell. It is transported into the plant cell against the concentration gradient via K^+^ transporter and membrane channels. High affinity K^+^ uptake mechanisms are mediated by K^+^ transporters when the extracellular K^+^ concentration is low, whereas low affinity uptake is carried out by K^+^ channels when the extracellular K^+^ concentration is high. Thus uptake mechanism is primarily determined by the concentration of K^+^ available in the soil. On the other hand a very low concentration of Na^+^ ion (about 1 mM or less) is maintained in the cytosol. During salinity stress, due to increased concentration of Na^+^ in the soil, Na^+^ ion competes with K^+^ for the transporter as they both share the same transport mechanism, thereby decreasing the uptake of K^+^ [3, 35].

A large number of genes and proteins, such as HKT and NHX, encoding K^+^ transporters and channels have been identified and cloned in various plant species. During salt stress expression of some low abundance transcripts is enhanced which are found to be involved in K^+^ uptake. This was observed in the halophyte* Mesembryanthemum crystallinum* [37]. Transporters located on the plasma membrane, belonging to the HKT (histidine kinase transporter) family, also play an essential role in salt tolerance by regulating transportation of Na^+^ and K^+^. Class 1 HKT transporters, that have been identified in* Arabidopsis,* protect the plant from the adverse effects of salinity by preventing excess accumulation Na^+^ in leaves. Similar results were observed in the experiment which was carried out with rice where class 1 HKT transporter removes excess Na^+^ from xylem, thus protecting the photosynthetic leaf tissues from the toxic effect of Na^+^ [38]. Intracellular NHX proteins are Na^+^, K^+^/H^+^ antiporters involved in K^+^ homeostasis, endosomal pH regulation, and salt tolerance. Barragán et al. [39] showed that tonoplast-localized NHX proteins (NHX1 and NHX2: the two major tonoplast-localized NHX isoforms) are essential for active K^+^ uptake at the tonoplast, for turgor regulation, and for stomatal function. In fact more such NHX isoforms have been identified and their roles in ion (Na^+^, K^+^, H^+^) homeostasis established from different plant species (e.g., LeNHX3 and LeNHX4 from tomato) [40].

### 2.2. Compatible Solute Accumulation and Osmotic Protection

Compatible solutes, also known as compatible osmolytes, are a group of chemically diverse organic compounds that are uncharged, polar, and soluble in nature and do not interfere with the cellular metabolism even at high concentration. They mainly include proline [41–45], glycine betaine [46, 47], sugar [48, 49], and polyols [50–53]. Organic osmolytes are synthesised and accumulated in varying amounts amongst different plant species. For example, quaternary ammonium compound beta alanine betaine's accumulation is restricted among few members of Plumbaginaceae [54], whereas accumulation of amino acid proline occurs in taxonomically diverse sets of plants [53]. The concentration of compatible solutes within the cell is maintained either by irreversible synthesis of the compounds or by a combination of synthesis and degradation. The biochemical pathways and genes involved in these processes have been thoroughly studied. As their accumulation is proportional to the external osmolarity, the major functions of these osmolytes are to protect the structure and to maintain osmotic balance within the cell via continuous water influx [24].

Amino acids such as cysteine, arginine, and methionine, which constitute about 55% of total free amino acids, decrease when exposed to salinity stress, whereas proline concentration rises in response to salinity stress [55]. Proline accumulation is a well-known measure adopted for alleviation of salinity stress [53, 56, 57]. Intracellular proline which is accumulated during salinity stress not only provides tolerance towards stress but also serves as an organic nitrogen reserve during stress recovery. Proline is synthesised either from glutamate or ornithine. In osmotically stressed cell glutamate functions as the primary precursor. The biosynthetic pathway comprises two major enzymes, pyrroline carboxylic acid synthetase and pyrroline carboxylic acid reductase. Both these regulatory steps are used to overproduce proline in plants [35]. It functions as an O_2_ quencher thereby revealing its antioxidant capability. This was observed in a study carried out by Matysik et al. [56]. Ben Ahmed et al. [57] observed that proline supplements enhanced salt tolerance in olive (*Olea europaea*) by amelioration of some antioxidative enzyme activities, photosynthetic activity, and plant growth and the preservation of a suitable plant water status under salinity conditions. It has been reported that proline improves salt tolerance in* Nicotiana tabacum* by increasing the activity of enzymes involved in antioxidant defence system [58]. Deivanai et al. [59] also demonstrated that rice seedlings from seeds pretreated with 1 mM proline exhibited improvement in growth during salt stress.

Glycine betaine is an amphoteric quaternary ammonium compound ubiquitously found in microorganisms, higher plants and animals, and is electrically neutral over a wide range of pH. It is highly soluble in water but also contains nonpolar moiety constituting 3-methyl groups. Because of its unique structural features it interacts both with hydrophobic and hydrophilic domains of the macromolecules, such as enzymes and protein complexes. Glycine betaine is a nontoxic cellular osmolyte that raises the osmolarity of the cell during stress period; thus it plays an important function in stress mitigation. Glycine betaine also protects the cell by osmotic adjustment [60], stabilizes proteins [61], and protects the photosynthetic apparatus from stress damages [62] and reduction of ROS [52, 53]. Accumulation of glycine betaine is found in a wide variety of plants belonging to different taxonomical background. Glycine betaine is synthesised within the cell from either choline or glycine. Synthesis of glycine betaine from choline is a 2-step reaction involving two or more enzymes. In the first step choline is oxidised to betaine aldehyde which is then again oxidised in the next step to form glycine betaine. In higher plants the first conversion is carried out by the enzyme choline monooxygenase (CMO), whereas the next step is catalysed by betaine aldehyde dehydrogenase (BADH) [63]. Another pathway which is observed in some plants, mainly halophytic, demonstrated the synthesis of glycine betaine from glycine. Here glycine betaine is synthesized by three successive N-methylation and the reactions are catalysed by two S-adenosyl methionine dependent methyl transferases, glycine sarcosine N-methyl transferase (GSMT), and sarcosine dimethylglycine N-methyl transferase (SDMT). These two enzymes have overlapping functions as GSMT catalyses the first and the second step while SDMT catalyses the second and third step [63]. Rahman et al. [64] reported the positive effect of glycine betaine on the ultrastructure of* Oryza sativa* seedlings when exposed to salt stress. Under stressed condition (150 mM NaCl) the ultrastructure of the seedling shows several damages such as swelling of thylakoids, disintegration of grana and intergranal lamellae, and disruption of mitochondria. However, these damages were largely prevented when seedlings were pretreated with glycine betaine. When glycine betaine is applied as a foliar spray in a plant subjected to stress, it led to pigment stabilization and increase in photosynthetic rate and growth [62, 63].

Polyols are compounds with multiple hydroxyl functional groups available for organic reactions. Sugar alcohols are a class of polyols functioning as compatible solutes, as low molecular weight chaperones, and as ROS scavenging compounds [52]. They can be classified into two major types, cyclic (e.g., pinitol) and acyclic (e.g., mannitol). Mannitol synthesis is induced in plants during stressed period via action of NADPH dependent mannose-6-phosphate reductase. These compatible solutes function as a protector or stabilizer of enzymes or membrane structures that are sensitive to dehydration or ionically induced damage. It was found that the transformation with bacterial* mltd* gene that encodes for mannitol-1-phosphate dehydrogenase in both* Arabidopsis* and tobacco (*Nicotiana tabacum*) plants confer salt tolerance, thereby maintaining normal growth and development when subjected to high level of salt stress [65, 66]. Pinitol is accumulated within the plant cell when the plant is subjected to salinity stress. The biosynthetic pathway consists of two major steps, methylation of myo-inositol which results in formation of an intermediate compound, ononitol, which undergoes epimerization to form pinitol. Inositol methyl transferase enzyme encoded by* imt* gene plays major role in the synthesis of pinitol. Transformation of* imt* gene in plants shows a result similar to that observed in the case of* mltd* gene. Thus it can be said that pinitol also plays a significant role in stress alleviation. Accumulation of polyols, either straight-chain metabolites such as mannitol and sorbitol or cyclic polyols such as myo-inositol and its methylated derivatives, is correlated with tolerance to drought and/or salinity, based on polyol distribution in many species, including microbes, plants, and animals [49].

Accumulations of carbohydrates such as sugars (e.g., glucose, fructose, fructans, and trehalose) and starch occur under salt stress [67]. The major role played by these carbohydrates in stress mitigation involves osmoprotection, carbon storage, and scavenging of reactive oxygen species. It was observed that salt stress increases the level of reducing sugars (sucrose and fructans) within the cell in a number of plants belonging to different species [48]. Besides being a carbohydrate reserve, trehalose accumulation protects organisms against several physical and chemical stresses including salinity stress. They play an osmoprotective role in physiological responses [63]. Sucrose content was found to increase in tomato (*Solanum lycopersicum*) under salinity due to increased activity of sucrose phosphate synthase [68]. Sugar content, during salinity stress, has been reported to both increase and decrease in various rice genotype [69]. In rice roots it has been observed that starch content decreased in response to salinity while it remained fairly unchanged in the shoot. Decrease in starch content and increase in reducing and nonreducing sugar content were noted in leaves of* Bruguiera parviflora* [67].

### 2.3. Antioxidant Regulation of Salinity Tolerance

Abiotic and biotic stress in living organisms, including plants, can cause overflow, deregulation, or even disruption of electron transport chains (ETC) in chloroplasts and mitochondria. Under these conditions molecular oxygen (O_2_) acts as an electron acceptor, giving rise to the accumulation of ROS. Singlet oxygen (^1^O_2_), the hydroxyl radical (OH^−^), the superoxide radical (O^−^_2_), and hydrogen peroxide (H_2_O_2_) are all strongly oxidizing compounds and therefore potentially harmful for cell integrity [70]. Antioxidant metabolism, including antioxidant enzymes and nonenzymatic compounds, play critical parts in detoxifying ROS induced by salinity stress. Salinity tolerance is positively correlated with the activity of antioxidant enzymes, such as superoxide dismutase (SOD), catalase (CAT), glutathione peroxidise (GPX), ascorbate peroxidase (APX), and glutathione reductase (GR) and with the accumulation of nonenzymatic antioxidant compounds [71, 72]. Gill et al. [73] and Tuteja et al. [74] have recently reported a couple of helicase proteins (e.g., DESD-box helicase and OsSUV3 dual helicase) functioning in plant salinity tolerance by improving/maintaining photosynthesis and antioxidant machinery. Kim et al. [75] showed that silicon (Si) application to rice root zone influenced the hormonal and antioxidant responses under salinity stress. The results showed that Si treatments significantly increased rice plant growth compared to controls under salinity stress. Si treatments reduced the sodium accumulation resulting in low electrolytic leakage and lipid peroxidation compared to control plants under salinity stress. Enzymatic antioxidant (catalase, peroxidase, and polyphenol oxidase) responses were more pronounced in control plants than in Si-treated plants under salinity stress.

Anthocyanin is a flavonoid whose accumulation in plant exposed to salt stress has been largely documented. Van Oosten et al. [76] isolated the* anthocyanin*-*impaired*-*response*-*1* (*air1*) mutant that is unable to accumulate anthocyanins under salt stress. The* air1* mutant showed a defect in anthocyanin production in response to salt stress but not to other stresses such as high light, low phosphorous, high temperature, or drought stress. This specificity indicated that* air1* mutation did not affect anthocyanin biosynthesis but rather its regulation in response to salt stress. The discovery and characterization of AIR1 opens avenues to dissect the connections between abiotic stress and accumulation of antioxidants in the form of flavonoids and anthocyanins.

Ascorbate is one of the major antioxidants present within the cell. Pea plants grown under saline (150 mM NaCl) stress showed an enhancement of both APX activity and S-nitrosylated APX, as well as an increase of H_2_O_2_, NO, and S-nitrosothiol (SNO) content that can justify the induction of the APX activity. Proteomic data have shown that APX is one of the potential targets of PTMs mediated by NO-derived molecules [77]. Using recombinant pea cytosolic APX, the impact of peroxynitrite (ONOO^−^) and S-nitrosoglutathione (GSNO), which are known to mediate protein nitration and S-nitrosylation processes, respectively, was analysed. While peroxynitrite inhibits APX activity, GSNO enhances its enzymatic activity. The results provide new insight into the molecular mechanism of the regulation of APX, which can be both inactivated by irreversible nitration and activated by reversible S-nitrosylation [77]. Exogenous application of ascorbate mitigates the adverse effects of salinity stress in various plant species and promotes plant recovery from the stress [78, 79]. Another antioxidant in stress mitigation is glutathione, which can react with superoxide radical, hydroxyl radical, and hydrogen peroxide, thereby functioning as a free radical scavenger. It can also participate in the regeneration of ascorbate via ascorbate-glutathione cycle [80]. When applied exogenously glutathione helped to maintain plasma membrane permeability and cell viability during salinity stress in* Allium cepa* [81]. Application of glutathione and ascorbate was found to be effective in increasing the height of the plant, branch number, fresh and dry weight of herbs and flowers, and the content of carbohydrates, phenols, xanthophylls pigment, and mineral ion content when subjected to saline condition [82]. Many studies have found differences in levels of expression or activity of antioxidant enzymes; these differences are sometimes associated with the more tolerant genotype and sometimes with the more sensitive genotype. Munns and Tester [3] suggested that differences in antioxidant activity between genotypes may be due to genotypic differences in degrees of stomatal closure or in other responses that alter the rate of CO_2_ fixation and differences that bring into play the processes that avoid photoinhibition and for which the plant has abundant capacity [3]. Roy et al. [83] in their recent review have argued that there are three main traits in plants, which help them in their adaptation to salinity stress: ion exclusion, tissue tolerance, and salinity tolerance. It seems that antioxidants have some role in tissue and salinity tolerance mechanism.

### 2.4. Roles of Polyamines in Salinity Tolerance

Polyamines (PA) are small, low molecular weight, ubiquitous, polycationic aliphatic molecules widely distributed throughout the plant kingdom. Polyamines play a variety of roles in normal growth and development such as regulation of cell proliferation, somatic embryogenesis, differentiation and morphogenesis, dormancy breaking of tubers and seed germination, development of flowers and fruit, and senescence [84–87]. It also plays a crucial role in abiotic stress tolerance including salinity and increases in the level of polyamines are correlated with stress tolerance in plants [88–91].

The most common polyamines that are found within the plant system are diamine putrescine (PUT), triamine spermidine (SPD), and tetra-amine spermine (SPM) [92–96]. The PA biosynthetic pathway has been thoroughly investigated in many organisms including plants and has been reviewed in details [97–104]. PUT is the smallest polyamine and is synthesised from either ornithine or arginine by the action of enzyme ornithine decarboxylase (ODC) and arginine decarboxylase (ADC), respectively [85, 105]. N-carbamoyl-putrescine is converted to PUT by the enzyme N-carbamoyl-putrescine aminohydrolase [106, 107]. The PUT thus formed functions as a primary substrate for higher polyamines such as SPD and SPM biosynthesis. The triamine SPD and tetramine SPM are synthesized by successive addition of aminopropyl group to PUT and SPD, respectively, by the enzymes spermidine synthase (SPDS) and spermine synthase (SPMS) [108, 109]. ODC pathway is the most common pathway for synthesis of polyamine found in plants. Most of the genes involved in the ODC pathway have been identified and cloned. However there are some plants where ODC pathway is absent; for instance in* Arabidopsis* polyamines are synthesized via ADC pathway [110–112]. All the genes involved in polyamine biosynthesis pathways have been identified from different plant species including* Arabidopsis* [113–115]. Polyamine biosynthesis pathway in* Arabidopsis* involves six major enzymes: ADC encoding genes (*ADC1* and* ADC2*); SPDS (*SPDS1* and* SPDS2*) and SAMDC (*SAMDC1, SAMDC2, SAMDC3, SAMDC4*) [115–118]. On the contrary, SPM synthase, thermospermine synthase, agmatine iminohydrolase and N-carbamoylputrescine amidohydrolase are represented by single genes only [119, 120].

Increase in endogenous polyamine level has been reported when the plant is exposed to salinity stress. Intracellular polyamine level is regulated by polyamine catabolism. Polyamines are oxidatively catabolised by amine oxidases which include copper binding diamine oxidases and FAD binding polyamine oxidases. These enzymes play a significant role in stress tolerance [121, 122]. The changes in cellular polyamine level due to stress provide possible implications in stress but do not provide evidence of their role in counteracting stress. Hence, to understand whether polyamines actually protect cells from stress-induced damages, exogenous application of polyamines, which is expected to increase endogenous polyamine, has been investigated before or during stress [123, 124]. Application of exogenous polyamine has been found to increases the level of endogenous polyamine during stress; the positive effects of polyamines have been associated with the maintenance of membrane integrity, regulation of gene expression for the synthesis of osmotically-active solutes, reduction in ROS production, and controlling accumulation of Na^+^ and Cl^−^ ion in different organs [123–130]. It was observed that plant deficient in* ADC1* and* ADC2* is hypersensitive to stress [131]. In* Arabidopsis*, expression of* ADC* and* SPMS* increases when exposed to salinity stress. whereas mutants of polyamine biosynthetic genes show sensitivity to salinity [132]. Overproduction of PUT, SPD, and SPM in rice, tobacco, and* Arabidopsis* enhances salt tolerance [133]. Salt stress regulates polyamine biosynthesis and catabolism by acting as a cellular signal in hormonal pathways thereby regulating abscisic acid (ABA) in response to stress [134]. Additionally, SPM and SPD are regarded as potent inducers of NO which is another important signalling molecule [135] and its involvement in salinity tolerance is discussed below. It has been reported that exogenous application of polyamines could alleviate salt-induced reduction in photosynthetic efficiency, but this effect depends on polyamine concentration and types and level of stress [136]. When the seedling of* Sorghum bicolor* treated with 0.25 mM SPM is subjected to salt stress it shows improvement in growth and partial increase in the activity of peroxidase and glutathione reductase enzyme with a concomitant decrease in the level of membrane lipid peroxidation [137]. Li et al. [138] performed 2-DE gel electrophoresis and MALDI-TOF/TOF MS with cytosolic proteins to understand the effect of exogenous SPD on proteomic changes under normal and NaCl stress of 3 days old cucumber seedling leaves. Many changes were observed in the levels of proteins involved in energy and metabolic pathways, protein metabolic, stress defense, and other functional proteins. They observed that increased salt tolerance by exogenous SPD would contribute to higher expressions of proteins involved in the SAMs metabolism, protein biosynthesis, and defense mechanisms on antioxidant and detoxification. Li et al. [138] also argued that the regulation of Calvin cycle, protein folding assembly, and the inhibition of protein proteolysis by SPD might play important roles in salt tolerance.

### 2.5. Roles of Nitric Oxide in Salinity Tolerance

Nitric oxide (NO) is a small volatile gaseous molecule, which is involved in the regulation of various plant growth and developmental processes, such as root growth, respiration, stomata closure, flowering, cell death, seed germination and stress responses, as well as a stress signalling molecule [139–143]. NO directly or indirectly triggers expression of many redox-regulated genes. NO reacts with lipid radicals thus preventing lipid oxidation, exerting a protective effect by scavenging superoxide radical and formation of peroxynitrite that can be neutralised by other cellular processes. It also helps in the activation of antioxidant enzymes (SOD, CAT, GPX, APX, and GR) [144].

Exogenous NO application has been found to play roles in stress mitigation [145–147], but the effects depend on NO concentration. Exogenous application of sodium nitroprusside (SNP), a NO donor, on* Lupinus luteus* seedlings subjected to salt stress enhanced seed germination and root growth [148]. Seed germination was promoted at concentrations between 0.1 and 800 *μ*M SNP in a dose-dependent manner. The stimulation was most pronounced after 18 and 24 h and ceased after 48 h of imbibition. The promoting effect of NO on seed germination persisted even in the presence of heavy metals (Pb and Cd) and NaCl. Kopyra and Gwóźdź [148] further showed that the pretreatment of* L. luteus* seedlings for 24 h with 10 *μ*M SNP resulted in efficient reduction of the detrimental effect of the abiotic stressors on root growth and morphology. Pretreatment of maize seedlings with 100 *μ*M SNP increases dry matter of roots and shoots under salinity stress; however, when the concentration of SNP was increased to 1000 *μ*M shoot and root dry weight decreased [149]. Thus, this experiment highlighted both the protective effects of low NO concentration and the toxic effect of high NO concentration on plants.

The positive effects of NO on salinity tolerance or stress mitigation have been attributed to antioxidant activities and modulation of ROS detoxification system [150]. Improved plant growth under salinity stress by exogenous application of NO was associated with increases in antioxidant enzymes such as SOD, CAT, GPX, APX, and GR [151], and suppression of malondialdehyde (MDA) production or lipid peroxidation [152]. Effects of NO on salinity tolerance are also related to its regulation of plasma membrane H^+^-ATPase and Na^+^/K^+^ ratio [143]. NO stimulates H^+^-ATPase (H^+^-PPase), thereby producing a H^+^ gradient and offering the force for Na^+^/H^+^ exchange. Such an increase of Na^+^/H^+^ exchange may contribute to K^+^ and Na^+^ homeostasis [149]. Although NO acts as a signal molecule under salt stress and induces salt resistance by increasing PM H^+^-ATPase activity, research results from Zhang et al. [153] with calluses from* Populus euphratica* also indicated NO cannot activate purified PM H^+^-ATPase activity, at least in vitro. They initially hypothesized ABA or H_2_O_2_ might be downstream signal molecules to regulate the activity of PM H^+^-ATPase. Further results indicated H_2_O_2_ content increased greatly under salt stress. Since H_2_O_2_ might be the candidate downstream signal molecule, Zhang et al. [153] tested PM H^+^-ATPase activity and K to Na ratio in calluses by adding H_2_O_2_. The results suggested that H_2_O_2_ inducing an increased PM H^+^-ATPase activity resulted in an increased K to Na ratio leading to NaCl stress adaptation.

### 2.6. Hormone Regulation of Salinity Tolerance

ABA is an important phytohormone whose application to plant ameliorates the effect of stress condition(s). It has long been recognized as a hormone which is upregulated due to soil water deficit around the root. Salinity stress causes osmotic stress and water deficit, increasing the production of ABA in shoots and roots [154–158]. The accumulation of ABA can mitigate the inhibitory effect of salinity on photosynthesis, growth, and translocation of assimilates [158, 159]. The positive relationship between ABA accumulation and salinity tolerance has been at least partially attributed to the accumulation of K^+^, Ca^2+^ and compatible solutes, such as proline and sugars, in vacuoles of roots, which counteract with the uptake of Na^+^ and Cl^−^ [160, 161]. ABA is a vital cellular signal that modulates the expression of a number of salt and water deficit-responsive genes. Fukuda and Tanaka [162] demonstrated the effects of ABA on the expression of two genes,* HVP1* and* HVP10*, for vacuolar H^+^-inorganic pyrophosphatase, and of* HvVHA-A,* for the catalytic subunit (subunit A) of vacuolar H^+^-ATPase in* Hordeum vulgare* under salinity stress. ABA treatment in wheat induced the expression of MAPK4-like, TIP 1, and GLP 1 genes under salinity stress [163].

Some other compounds having hormonal properties, such as salicylic acid (SA) and brassinosteroids (BR), also participate in plant abiotic stress responses [164, 165]. Under salinity stress endogenous level of SA increased along with the increase in the activity of salicylic acid biosynthetic enzyme in rice seedling [166]. Jayakannan et al. [167] have recently shown that SA improves salinity tolerance in* Arabidopsis* by restoring membrane potential and preventing salt-induced K^+^ loss via a guard cell outward rectifying K(+) (GORK) channel.* Arabidopsis* seedling pretreated with SA showed upregulation of H^+^-ATPase activity, thereby improving K^+^ retention during salt stress; SA pretreatment did not prevent accumulation of Na^+^ in roots but somehow helped to reduce the concentration of accumulated Na^+^ in the shoot [167]. The application of SA also promoted salinity tolerance in barley, as manifested by increases in the content of chlorophyll and carotenoid and maintaining membrane integrity, which was associated with more K^+^ and soluble sugar accumulation in the root under saline condition [168]. Nazar et al. [169] have argued that SA alleviates decreases in photosynthesis under salt stress by enhancing nitrogen and sulfur assimilation and antioxidant metabolism differentially in mung bean cultivars. The negative effects of salinity may also be mitigated by BR [170–173]. Application of BR enhanced the activity of antioxidant enzymes (SOD, POX, APX, and GPX) and the accumulation of nonenzymatic antioxidant compounds (tocopherol, ascorbate, and reduced glutathione) [170]. Both BRs and SA are ubiquitous in the plant kingdom, affecting plant growth and development in many different ways, and are known to improve plant stress tolerance. Ashraf et al. [173] have reviewed and discussed the current knowledge and possible applications of BRs and SA that could be used to mitigate the harmful effects of salt stress in plants. They have also discussed the roles of exogenous applications of BRs and SA in the regulation of various biochemical and physiological processes leading to improved salt tolerance in plants.

## 3. Transcriptional Regulation and Gene Expression of Salinity Tolerance

Regulation of gene expression in salinity stress includes a wide array of mechanisms that are used by plants to upregulate or downregulate (increase or decrease) the production of specific gene products (protein or RNA). Various mechanisms of gene regulation have been identified during the central dogma, from transcriptional initiation, to RNA processing, and to the posttranslational modification of a protein.

Transcriptomic analysis provides detailed knowledge about the gene expression at mRNA level, which is widely used to screen candidate genes involved in stress responses. Genomic approaches play a significant role in encoding, cloning, and characterization of important genes. A huge number of salt-responsive transcription factors and genes which are either upregulated or downregulated in response to salinity stress have been identified and characterized using transcriptomic and genomic approaches.

Transcription factors are considered as most important regulators that control gene expressions. Among them, bZIP, WRKY, AP2, NAC, C2H2 zinc finger gene, and DREB families comprise a large number of stress-responsive members. These transcription factor genes are capable of controlling the expression of a broad range of target genes by binding to the specific cis-acting element in the promoters of these genes. Johnson et al. [174] observed that the expression of bZIP genes were upregulated in salt-sensitive wheat cultivar, when exposed to long-term salinity, but decreased in salt-tolerant variety. Overexpression of a NAC transcription factor in both rice and wheat confers salt tolerance, thereby predicting their role in stress mitigation [175]. In rice transcriptional regulators that have been demonstrated to play a significant role in abiotic stress responses involve DREB1/CBF, DREB2, and AREB/ABF [176–180]. Transcriptions factors such as OsNAC5 and ZFP179 show an upregulation under salinity stress, which may regulate the synthesis and accumulation of proline, sugar, and LEA proteins that in turn play an integral role in stress tolerance [181]. In* Arabidopsis*, salt stress results in the upregulation of AtWRKY8 which directly binds with the promoter of* RD29A*, suggesting it to be as one of the target genes of AtWRKY8 [182].

A large number of genes and transcription factors are upregulated in response to salinity in different plant species, which serve diverse functions [183–192]. Examples of salt-responsive genes are listed in the Table 1, and these genes are mainly classified into the following functional categories: ion transport or homeostasis (e.g.,* SOS* genes,* AtNHX1*, and* H* ^*+*^ *-ATPase*), senescence-associated genes (e.g.,* SAG*), molecular chaperones (e.g.,* HSP* genes), and dehydration-related transcription factors (e.g.,* DREB*). Among stress-responsive genes, the* SOS* gene family, which we have already discussed in Section 2.1, is believed to play a very intriguing role in ion homeostasis, thereby conferring salt tolerance [24–37, 190, 191]. Some ROS-scavenging and osmotic-regulating genes are also upregulated by salinity in some plant species. For example, a continuous exposure of rice plants to salinity for about 24 hours resulted in upregulation of glutathione-S-transferase and ascorbate peroxidase, both of which were known to play an active role in ROS scavenging, and with the increase in duration of exposure to salinity stress, upregulation of metallothionein and water channel proteins was also observed [192]. Halophyte plant species* Spartina alterniflora* when subjected to salt stress exhibits upregulation of 10 genes associated with osmotic regulation [193].

Recently, Schmidt et al. [194] identified* SALT-RESPONSIVE ERF1 (SERF1)*, a rice (*Oryza sativa*) transcription factor gene that showed a root-specific induction upon salt and H_2_O_2_ treatment. Loss of* SERF1* impaired the salt-inducible expression of genes encoding members of a mitogen-activated protein kinase (MAPK) cascade and salt tolerance-mediating TFs. Furthermore, they showed that SERF1-dependent genes are H_2_O_2_ responsive and demonstrated that SERF1 binds to the promoters of* MAPK KINASE KINASE6 (MAP3K6), MAPK5, DEHYDRATION-RESPONSIVE ELEMENT BINDING2A (DREB2A),* and* ZINC FINGER PROTEIN179 (ZFP179)* in vitro and in vivo. SERF1 also directly induces its own gene expression. In addition, it was observed that SERF1 is a phosphorylation target of MAPK5, resulting in enhanced transcriptional activity of SERF1 toward its direct target genes. Finally, they demonstrated that the plants deficient for SERF1 are more sensitive to salt stress compared with the wild type, while constitutive overexpression of SERF1 improves salinity tolerance.

There are some transcription factors which are regulated by different kinases and have been found to be significant players of plant adaptation to salinity stress. Serra et al. [195] showed that* OsRMC* encodes a receptor-like kinase described as a negative regulator of salt stress responses in rice. Two transcription factors, OsEREBP1 and OsEREBP2, belonging to the AP2/ERF family were shown to bind to the same GCC-like DNA motif in* OsRMC* promoter and to negatively regulate its gene expression. Serra et al. [195] further revealed that* OsEREBP1* transcript level is not significantly affected by salt, ABA, or severe cold (5°C) and is only slightly regulated by drought and moderate cold. On the other hand, the* OsEREBP2* transcript level increased after cold, ABA, drought, and high salinity treatments, indicating that OsEREBP2 may play a central role mediating the response to different abiotic stresses. Gene expression analysis in rice varieties with contrasting salt tolerance further suggests that* OsEREBP2* is involved in salt stress response in rice. A bZIP class of ABRE binding transcription factor, known as OSBZ8, has also been identified from rice and has been shown to be highly expressed in salt tolerant cultivars than in salt sensitive one [196]. Moreover, OSBZ8 has been shown to be activated/phosphorylated by a SNF-1 group of serine/threonine kinase in the presence of Spd during salinity stress [197].

High-throughput sequencing for transcript profiling in plants has revealed that alternative splicing affects a much higher proportion of the transcriptome than was previously assumed. Alternative splicing is involved in most plant processes and is particularly prevalent in plants exposed to environmental stress. The identification of mutations in predicted splicing factors and spliceosomal proteins that affect cell fate, the circadian clock, plant defense, and tolerance/sensitivity to abiotic stress all points to a fundamental role of splicing/alternative splicing in plant growth, development, and responses to external cues [198]. A suite of Ser/Arg-rich proteins that are key regulators of alternative splicing undergoes alternative themselves in response to various abiotic stresses, such as salt stress [198–200]. PRMT5, a type II protein Arg methyltransferase that symmetrically dimethylates Arg side chains, also impacts splicing/alternative in Arabidopsis. The prmt5 mutant, also known as shk1 kinase binding protein1 (skb1), is sensitive to salt [201]. It was proposed that PRMT5/SKB1 affects plant development and the salt response by altering the methylation status of H4R3sme2 (for symmetric dimethylation of histone H4 arginine 3) and LSm4 and thus linking transcription to pre-mRNA splicing [201]. A nuclear coactivator, At-SKIP (Ski-interacting protein), expression was found to increase in response to salt, mannitol, and ABA treatment, and At-SKIP overexpression or antisense lines show altered tolerance to a plethora of abiotic stress factors [186], and it is likely that a role in alternative splicing contributes to these phenotypes [198].

The small ubiquitin-like modifier (SUMO) is a crucial regulator of signaling proteins in eukaryotes. Attachment of SUMO onto substrates is reversible, and SUMO proteases, which specifically cleave the SUMO-substrate linkages, play a vital regulatory role during SUMOylation. Conti et al. [202] have identified two SUMO proteases, Overly Tolerant To Salt1 (OTS1) and OTS2, which are localized in the nucleus and act redundantly to regulate salt stress responses in* Arabidopsis thaliana*. Cui et al. [203] have identified an* Arabidopsis* endoplasmic reticulum (ER-) associated protein degradation (ERAD) component called Ubiquitin conjugase UBC32 that functions in BR-mediated salt stress tolerance. More and more such reports of sumoylation and other ubiquitin like posttranslational modifications during plant salinity stress are coming up.

Downregulated genes are emerging now as essential components of the response to salinity. For example downregulation of *β*-carotene hydroxylase increases *β*-carotene and total carotenoids enhancing salt stress tolerance in transgenic cultured cells of sweet potato [204]. It seems that mutual regulation mechanism exists between different genes and proteins and signals underlying different processes of plant adaptation to abiotic stress.

In addition to protein coding genes, recently discovered microRNAs (miRNAs) and endogenous small interfering RNAs (siRNAs) have emerged as important players in plant stress responses. Initial clues suggesting that small RNAs are involved in plant stress responses stem from studies showing stress regulation of miRNAs and endogenous siRNAs, as well as from target predictions for some miRNAs [205, 206].

## 4. Proteomic and Metabolic Responses to Salinity Stress

Genomics technologies have helped to address the multigenicity of the plant abiotic stress responses. Analysis of genome sequences, and specific transcript collections and their dynamic changes, has provided a more global picture of stress-dependent responses at the cell, tissue, and whole plant level and moved the field from a single-gene approach toward an understanding of interactions between multiple components in cells, facilitating the dissection of abiotic stress circuits and coexpression hubs [5–8, 207–209]. However, directly focusing on genes may not accurately portray conditions in the cell at a particular state and time during stress due to regulation at the RNA and protein level, including posttranslational regulation. Proteomics, and in particular quantitative proteomics, is emerging as a powerful technique to be applied to the field of crop abiotic stress tolerance research; it has the potential to allow rapid identification and quantification of novel stress- and tolerance-related proteins. Understanding the dynamics of expression and posttranslational modifications of these proteins, and gaining direct insight into their function and interactions, can provide essential information that can be applied to engineer stress-tolerant crops with novel traits through biomarker selection and transgenic strategies [209, 210]. Available data suggest that several common stress responsive proteins are expressed in response to various abiotic stresses in different plant species. About 2171 proteins from 34 plant species have been identified and characterized as salt-responsive proteins, which are either upregulated or downregulated by salinity stress [211]. Based on gene ontology, BLAST alignment and literature information, salt-responsive proteins can be grouped into 14 functional categories. Specifically, there appears to be a general regulation of proteins involved in carbohydrate, nitrogen, and energy metabolisms, with particular emphasis on glycolytic and Krebs cycle enzymes. Moreover, as discussed earlier, salinity and other abiotic stresses lead to metabolic imbalances that lead to ROS generation. Therefore, it is not surprising that plant root or shoot proteomics show the expression of ROS scavenging proteins like SOD, CAT, GPX, APX, and GR [209, 212]. Other proteins that are identified in multiple studies are those involved in protein synthesis, processing, turnover, and degradation, as well as cytoskeleton stability. For photosynthetic processes, there appears to be a general decrease in levels of chlorophyll biosynthesis related proteins but an increase in proteins involved in the light-dependent reactions. Some of the proteins identified are indicative of a general stress-responsive pathway in plants. Less common are proteins identified in the categories of signaling, trafficking, transport, and cell structure [209]. Plant lamina or root membrane proteomics, including that of plasma membrane, mitochondrial, and thylakoid membrane, have revealed the up-/downregulation of a plethora of proteins. These include receptor proteins that perceive the stress, membrane bound signaling, and regulatory proteins that function to relay the stress, vesicle trafficking proteins, and transport proteins that function to maintain ion and water homeostasis, and drive sequestration and/or removal of toxic compounds from the cell, membrane bound kinases, and intrinsic proteins [213–219].

Another significant research approach in plant system biology is the metabolomics which involves the study of metabolome. Higher plants have a remarkable ability to synthesize a vast array of metabolites that differ in chemical complexity and biological functions playing an indispensible role in stress alleviation [229, 230]. Examples of plant metabolites that are involved in salinity tolerance include polyols such as mannitol and sorbitol, dimethylsulfonium compounds, glycine betaine, sugars such as sucrose, trehalose and fructans, or amino acids such as proline that serve as an osmolyte or osmoprotectant [231]. Plants when subjected to salinity stress show an increase in the concentration of these osmolytes thus playing significant role in stress mitigation. We have already discussed their role in Section 2.2. Advances in analytical chemistry, such as MS based methods and NMR, and sophisticated “data processing and mining” techniques, have allowed the plant biologists to venture into hitherto unexplored domains and generate extensive metabolic profiles due to various environmental stimuli including salinity. Results indicate that the metabolic processes are highly specific for given tissues, species, and plant-environment interactions. The clusters of identified compounds not only serve as base in the quest of novel defense compounds but also as markers for the characterization of the plants' defensive state. The latter is especially useful in agronomic applications where meaningful markers are essential for crop protection [232].

## 5. Bioengineering for Improving Salinity Tolerance

Genetic transformation technology enables scientists to achieve gene transfer in precise and predictable manner. Hence genetic engineering approaches would be useful to manipulate the osmoprotectants biosynthetic pathways for accumulating such molecules that act by scavenging ROS, reducing lipid peroxidation, maintaining protein structure and functions [233–235]. Many works on the transformation of plants for improving salinity tolerance focus on genes controlling ion transport, as regulation of Na^+^ uptake and compartmentalization is a critically important mechanism for plant survival under salinity stress, and many candidate genes controlling this mechanism have been identified. Engineering plants for overexpression of genes encoding for antiporters is identified as an effective method for generating salt-tolerant plants (Table 2 and relative references in Table 2). Gene expression studies using constitutive promoters provide limited biological information compared with the use of inducible promoters or cell type-specific promoters. The choice of promoters can significantly affect the results from a transgenic manipulation. Thus salt tolerant crops could be engineered by (1) successful fine-tuning of the stress response by engineering novel regulatory targets; (2) proper understanding of posttranslational modifications which regulate plant growth performance under stress; (3) overexpression of miRNAs or their targets; (4) maintaining hormone homeostasis to avoid pleiotropic effects under stress; and (5) applying plant synthetic biology approaches to improve genetic engineering strategies [236].

## 6. Conclusions and Future Research Perspectives

Salinity tolerance involves a complex of responses at molecular, cellular, metabolic, physiological, and whole-plant levels. Extensive research through cellular, metabolic, and physiological analysis has elucidated that among various salinity responses, mechanisms or strategies controlling ion uptake, transport and balance, osmotic regulation, hormone metabolism, antioxidant metabolism, and stress signalling play critical roles in plant adaptation to salinity stress. Taking advantage of the latest advancements in the field of genomic, transcriptomic, proteomic, and metabolomic techniques, plant biologists are focusing on the development of a complete profile of genes, proteins, and metabolites responsible for different mechanisms of salinity tolerance in different plant species. However, there is lack of the integration of information from genomic, transcriptomic, proteomic, and metabolomics studies, and the combined approach is essential for the determination of the key pathways or processes controlling salinity tolerance. In addition, in spite of the significant progress in the understanding of plant stress responses, there is still a large gap in our knowledge of transmembrane ion transport, sensor and receptor in the signalling transduction, molecules in long distance signalling, and metabolites in energy supply. The future focus should be on the study of intercellular and intracellular molecular interaction involved in salinity stress response. Genetic engineering has been proved to be an efficient approach to the development of salinity-tolerant plants, and this approach will become more powerful as more candidate genes associated with salinity tolerance are identified and widely utilized.

## Figures and Tables

**Table 1:** Examples of upregulated genes in response to salinity stress.

Species	NaCl concentration (mM)	Gene name	Gene functions	References
*Brassica juncea* and *Brassica campestris *	25 and 50	*SOS1* *SOS2* *SOS3* *AtNHX1 *	(i) Plasma membrane Na^+^/K^+^ antiporter. (ii) Protein kinase. (iii) Calcium-binding protein. (iv) Vacuolar Na^+^/K^+^ antiporter.	[183]

*Oryza sativa *	50	*PRP* *SAG* *HSPC025 *	(i) Proline-rich proteins and cell-wall protection. (ii) Senescence associated genes, regulatory processes, and cellular signal transduction. (iii) Heat-shock proteins, protein stabilizing.	[184]

*Oryza sativa *	100	*OsHSP23.7 OsHSP71.1, OsHSP80.2 *	Heat-shock proteins, molecular chaperones, folding, assembling, and transporting proteins.	[185]

*Arabidopsis thaliana *	150	*AtSKIP *	Transcription factor, transcriptional pre-initiation, splicing, and polyadenylation.	[186]

*Oryza sativa *	200	*OsHsp17.0, OsHsp23.7 *	Heat-shock proteins, molecular chaperones, and folding, assembling, and transporting proteins.	[187]

Carrot	300	*DcHsp17.7 *	Cell viability and membrane stability under heat stress.	[188]

*Arabidopsis thaliana *	300	*JcDREB *	Transcription factor	[189]

**Table 2:** Improving plant salt tolerance through engineering genes for various membrane antiporters.

Transgenic host	Gene engineered	Source	Improved functions under salinity stress	References
Arabidopsis	Vacuolar Na^+^/H^+^ antiporter Ms NHX1	Alfalfa (*Medicago sativa*)	Increased osmotic balance. MDA content rises.	[220]

Rice	Vacuolar Na^+^/H^+^ Antiporter Pg NHX1	*Pennisetum glaucum *	Elaborate root system.	[221]

Wheat	Vacuolar Na^+^/H^+^ Antiporter At NHX1	*Arabidopsis thaliana* L.	Increase in grain yield and biomass production. Accumulation of K^+^ in leaf. Reduced aggregation of Na^+^.	[222]

Tobacco	Vacuolar Na^+^/H^+^ antiporter GhNHX1	*Gossypium hirsutum *	Na^+^ compartmentalization.	[223]

Tomato	Vacuolar Na^+^/H^+^ antiporter AtNHX1	*Arabidopsis thaliana* L.	Over production of vacuolar Na^+^/H^+^ antiporter.	[224]

Tobacco	Vacuolar Na^+^/H^+^ antiporter AlNHXI	*Aeluropus littoralis *	Compartmentalization of Na in roots. Maintenance of K^+^/Na^+^ ratio in the leaf.	[225]

Brassica	Vacuolar Na^+^/H^+^ antiporter AtNHX1	*Arabidopsis thaliana* L.	Increased proline content. Improved growth rate. Mitigate the toxic effect of Na^+^.	[226]

Arabidopsis	Plasma membrane Na^+^/H^+^ antiporter SOS1	*Arabidopsis thaliana* L. (wild type)	Improved germination rate, root growth, and chlorophyll content. Reduced accumulation of Na^+^.	[227]

Maize	Vacuolar Na^+^/H^+^ antiporter AtNHX1	*Arabidopsis thaliana* L.	Increased rate of germination.	[228]
